# Obtaining and Characterization of the PLA/Chitosan Foams with Antimicrobial Properties Achieved by the Emulsification Combined with the Dissolution of Chitosan by CO_2_ Saturation

**DOI:** 10.3390/molecules24244532

**Published:** 2019-12-11

**Authors:** Szymon Mania, Karolina Partyka, Joanna Pilch, Ewa Augustin, Mateusz Cieślik, Jacek Ryl, Jia-Rong Jinn, Ya-Jane Wang, Anna Michałowska, Robert Tylingo

**Affiliations:** 1 Department of Chemistry, Technology and Biotechnology of Food, Faculty of Chemistry, Gdansk University of Technology, 11/12 G. Narutowicza Street, 80-233 Gdansk, Poland; robertt@pg.edu.pl; 2 Chitone Sp. z o.o., 15 Pionierów Street, 84-351 Lębork, Poland; k.partyka@chitone.pl; 3 Department of Pharmaceutical Technology and Biochemistry, Faculty of Chemistry, Gdansk University of Technology, 11/12 G. Narutowicza Street, 80-233 Gdansk, Poland; joapilch@pg.edu.pl (J.P.); ewa.augustin@pg.edu.pl (E.A.); 4 Department of Electrochemistry, Corrosion and Material Engineering, Faculty of Chemistry, 11/12 G. Narutowicza Street, 80-233 Gdansk, Poland; matciesl2@student.pg.edu.pl (M.C.); jacek.ryl@pg.edu.pl (J.R.); 5 Department of Food Science, University of Arkansas, 2650 N. Young Ave., Fayetteville, AR 72704, USA; jinn@uark.edu (J.-R.J.); yjwang@uark.edu (Y.-J.W.); 6 AGC Biologics, Vandtårnsvej 83B, 2860 Søborg, Copenhagen, Denmark; anna.s.michalowska@gmail.com

**Keywords:** antimicrobial properties, chitosan, CO_2_ saturation, foam, PLA, PEG

## Abstract

A new method of obtaining functional foam material has been proposed. The materials were created by mixing the poly lactic acid (PLA) solution in chloroform, chitosan (CS) dissolved in water saturated with CO_2_ and polyethylene glycol (PEG), and freeze-dried for removal of the solvents. The composite foams were characterized for their structural (SEM, FT-IR, density, porosity), thermal (DSC), functional (hardness, elasticity, swelling capacity, solubility), and biological (antimicrobial and cytotoxic) properties. Chitosan in the composites was a component for obtaining their foamed form with 7.4 to 22.7 times lower density compared to the neat PLA and high porosity also confirmed by the SEM. The foams had a hardness in the range of 70–440 kPa. The FT-IR analysis confirmed no new chemical bonds between the sponge ingredients. Other results showed low sorption capacity (2.5–7.2 g/g) and solubility of materials (less than 0.2%). The obtained foams had the lower T_g_ value and improved ability of crystallization compared to neat PLA. The addition of chitosan provides the bacteriostatic and bactericidal properties against *Escherichia coli* and *Staphylococcus aureus*. Biocompatibility studies have shown that the materials obtained are not cytotoxic to the L929 cell line.

## 1. Introduction

Foams are one of the forms of the polymer constructs intensively developing in material engineering. The main factors responsible for their popularity are low weight, low density, and reduced costs of products based on them. This means that they are used in a wide range of functional materials. Currently, the polymer foam market is dominated by the conventional polymer foams made mostly of polystyrene (PS) and polypropylene (PP). However, in specialized industries such as the tissue engineering, it is necessary to use materials that, in addition to standard mechanical properties, have additional functions. These functions are often associated with the activation of growth of the bone tissue, cartilage, ligaments, skin, blood vessels, nerves, and muscles [1]. Spongy materials are also used as the carriers for the controlled drugs release [2]. Due to the origin of the raw material, spongy materials are divided into synthetic, natural, and ceramic materials and their combinations. Clearly, the natural polymers are seen to have the greatest potential in tissue engineering include collagen, the protein that forms the majority of the extracellular matrix, alginate-a plant polymer derived from the algae, and chitosan obtained from chitin and present mainly in protective shells of crustaceans [3,4]. The advantage of the synthetic materials is better control of chemical, physical, and mechanical properties. The most popular polymers in this group are linear aliphatic polyesters, which include polyglycolic acid (PGA), polylactic acid (PLA), and their copolymers (PLGA). Their degradation involves random hydrolysis of ester bonds, e.g., PLA breaks down into the lactic acid, which is present in the human body [5]. The spongy materials can also be created by combining synthetic and natural materials [6]. Natural fillers such as chitosan [7] and cellulose [8,9] can be used to improve the properties of PLA-based foams. Chitosan is obtained from chitin in a deacetylation process. Chitin is the second most common polysaccharide in nature after cellulose and is obtained from crustacean processing waste. Chitin, chitosan, and materials obtained from them are bioavailable, biocompatible, biodegradable, and biofunctional. Moreover, they do not have antigenic properties and are non-toxic and environmentally friendly [10,11]. The presence of two functional groups in the molecule of chitosan, i.e., hydroxyl and amine, makes it possible to carry out many chemical and enzymatic modifications. Thus, it is often used in the design and construction of systems for the immobilization and release of therapeutic compounds, as well as for obtaining water-soluble derivatives of chitosan [12,13]. The specific feature of chitosan is its antibacterial and antifungal activity [14]. These properties exhibit acidic chitosan solutions, hydrogel forms, films, and dry sponges [15,16,17]. The antimicrobial activity of chitosan depends on many physicochemical factors: the molecular weight of the polymer, its degree of deacetylation, the pH of the environment, and the changes caused by the modification. Scientists have not strictly defined the mechanism of chitosan antibacterial activity. According to one theory, the antimicrobial activity of chitosan is associated with its polycationic character and interactions with the negatively charged bacterial cell membrane. These interactions can lead to changes in the permeability of the cell membrane, thus causing an osmotic imbalance inside the cell, and consequently inhibiting the growth of microorganisms [18]. They may also be responsible for the hydrolysis of peptidoglycan in the bacterial cell wall, which leads to leakage of intracellular electrolytes [19,20]. Chitosan demonstration of antibacterial properties can probably also be caused by its ability to chelate metal ions necessary for the growth of microorganisms [18]. Additional modifications, e.g., with N-propyl phosphonic anhydride, also affect the intensification of antibacterial properties [13]. It can be assumed that the increased antibacterial activity of the modified chitosan is mainly associated with the solubility of the polymer in a neutral pH environment, its polycationic character, as well as the presence of phosphate groups that are responsible for chelation of cations. This promotes the formation of intermolecular and intramolecular hydrogen bonds, which, by the way, produce hydrophobic micro-spaces within the polymer chain. The local division of the polymer area into hydrophobic and hydrophilic fragments promotes, in structural terms, the affinity between the bacterial cell wall and the chitosan derivative [13,18]. One of the main limitations of combining chitosan as a carrier of antimicrobial activity with synthetic polymers such as PLA or PGA is its hydrophilic character. It is possible to chemically modify chitosan in such a way that it exhibits hydrophobic properties and interacts more strongly with PLA, giving new properties to the obtained biocomposites [21]. Another solution may be to use the technique of emulsification and lyophilization of polymer solutions of different chemical nature, which enables, for example, the obtaining of chitosan/collagen/PLA biocomposites [22]. Most techniques for obtaining PLA/CS composites are based on the use of a chitosan solution dissolved in diluted organic acids, which entails the use of additional methods associated with the removal or neutralization of acid residues in the finished material [23,24].

An alternative way may be to use the method of producing material in the following work by mixing a solution of PLA in chloroform, and chitosan precipitate dissolved in water saturated with CO_2_, and polyethylene glycol and freeze-drying to remove solvents: water and volatile chloroform. In the scientific literature are known the methods of using carbon dioxide for the preparation of similar materials. Hijazi et al. presented two supercritical CO_2_-assisted processes aimed at generating chitosan nanoparticles for modification of the PLA films [25]. The work of Kazimierczak et al. presents method of foaming materials by using chemically produced CO_2_ directly in the chitosan/agarose/nanohydroxyapatite scaffolds [26]. The novelty of our work is the use of carbonic acid in the construction of foamed PLA/CS composites, which by using lyophilization allows the obtaining of foamed materials in a simple way. The use of the CO_2_ saturation technique of chitosan has not yet been presented in the design of this type of functional materials. The obtained foams were characterized by a lack of cytotoxicity toward L929 fibroblast cells, low density, and high porosity and hardness, and possess antimicrobial activity, the effectiveness of which depends on the share of chitosan in the composite.

## 2. Results and Discussion

### 2.1. Structure and Physicochemical Properties

The chemo-physical properties of the fabricated foams were investigated. Obtaining the foamed form of neat PLA by the presented method was impossible. Only using the solution of chitosan dissolved by CO_2_ saturation of its precipitate suspension allows the creation of the foam form of the composite. The use of the polyethylene glycol stabilized the mixture of the PLA and chitosan against delamination until freezing. The density was an important parameter describing the physical properties of the material. This parameter had an influence on the mechanical and thermal properties [27]. It also had great importance from an economic point of view because the higher density means the higher cost of the final material, which could be adverse for potential customers. The density of polymer foam depends on the amount and density of the material making up its network, and the amount of gas trapped in foam during the process of its production [28]. The density of PLA/CS foam was significantly reduced compared to the PLA density, from 7.4 to 22.7 times with increasing content of the chitosan in foam (Table 1). For comparison, the density of the PLA and its derivatives such as poly(L-lactic acid) (PLLA) and poly(D-lactic acid) (PDLA) has been reported as 1210–1250 kg/m^3^, 1240–1300 kg/m^3^, and 1250–1270 g/m^3^ for the amorphous and crystalline form of the polymer, respectively [29]. In turn, the density of chitosan foam corresponds to the data obtained by Mathias and co-workers [30]. Both foams were obtained from a 1% chitosan solution. The addition of 4% chitosan made it possible to obtain a foamed form, which may indicate that chitosan forms a foam for the PLA.

The porosity and pore size at the macroscopic and the microscopic levels are important parameters for designing active susceptibility release systems or foam materials (scaffolds) for other biomedical applications. It is considered that for ensuring the adequate nutrient or gas exchange, the porosity of foams should be more than 80%. The porosity of the tested samples depended on their density. The PLA had the highest density and the smallest chitosan foam. Therefore, reducing the concentration of chitosan composite with the PLA resulted in an increase in foam density. The CS and CS16 samples were the most porous and met the criteria.

**Figure 1:**
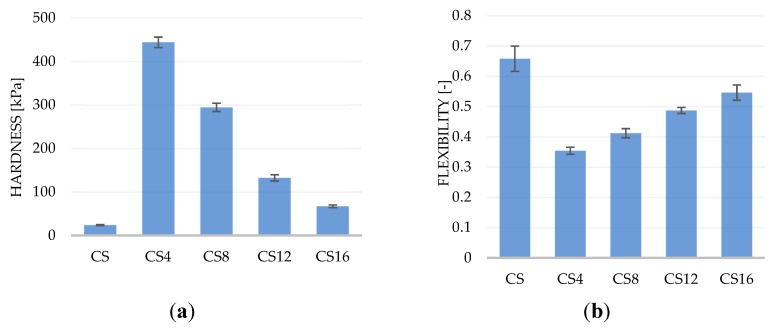
Mechanical properties: (**a**) hardness and (**b**) flexibility of the PLA/CS foams. Values represent means ± standard deviation (*n* = 3, *p* < 0.05).

The requirement of the mechanical properties of foams depends on the anatomic site for regeneration and the mechanical loads present at the site. An increase of porosity usually causes deterioration of the material′s mechanical properties [31]. The mechanical properties of the materials were compared based on two texture analysis parameters: hardness and elasticity. In this test, the sample was twice compressed. The result of the test was a graph of compression force versus time in which there were two peaks corresponding to the first and second sample compression cycles. The maximum force value recorded in the first compression cycle corresponded to the hardness of the sample. The flexibility was determined as the ratio of the time of the second deformation of the sample to the time of the first deformation of the sample, in which the compression was carried out up to 50% deformation relative to the initial height of the sample. The results of measuring the hardness and elasticity of the spongy materials are presented in Figure 1. The hardness of chitosan foam was the lowest (23.8 kPa) and corresponds to the hardness of similar materials created from a 1% solution which was described in our other works [3,32]. The hardest turned out to be foams with the lowest content of chitosan (CS4), and the increase in the content of this polysaccharide in the composite reduced the hardness of the materials. The foam with the highest chitosan content (CS16) was almost three times harder than the CS sample. For the flexibility, exactly the opposite relationship was observed. The most flexible was the chitosan foam and the least the CS4 composite foam. The increase in the chitosan content in the foam increased the elasticity of the composite. During compression of the porous material, a substantial part of the deformation was associated with deformation of the pores filled with air. Therefore, the results of mechanical tests also depend on the porosity of the materials. Based on the obtained results, it can be said that as the porosity increases, the hardness decreases, and the elasticity of composite foams increases. This relationship is not always so simple because the mechanical properties may be attributed to other factors, such as the orientation and relative positioning of the fibers along the material [33].

**Figure 2:**
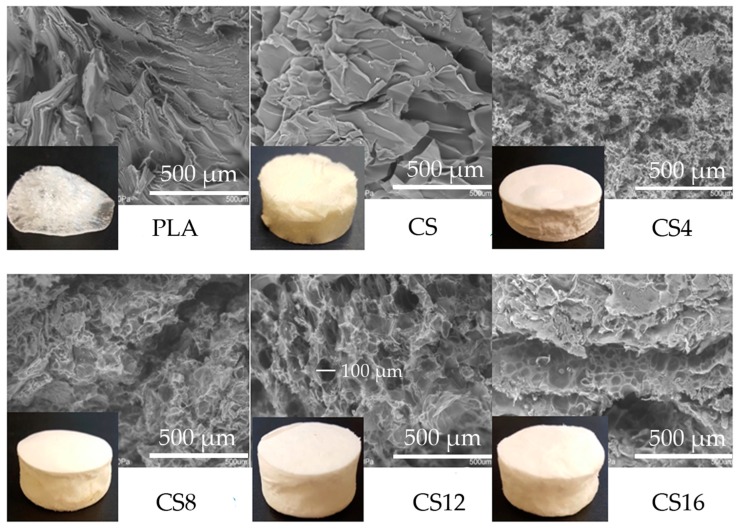
Comparison of the optical and scanning images of the obtained PLA/CS foams and control samples.

The method of saturating the solution with carbon dioxide gas was not applicable to foaming PLA from a chloroform solution, which is confirmed in Figure 2.

The PLA sample was rather a form of an irregular film. All foams had the color of the raw materials from which they were obtained, or it was the value resulting from the share of foam components. The porous structure was best emphasized in the case of composite foams. The microstructure of the materials shown in Figure 2 may suggest that the porosity of the chitosan foam should not be the largest or comparable with the CS16 sample. However, the smallest density and hardness of CS sample caused difficulty with perfect cutting without its compression. The pore regularity was noted only for the CS12 sample, which had a diameter of approximately 100 μm.

**Figure 3:**
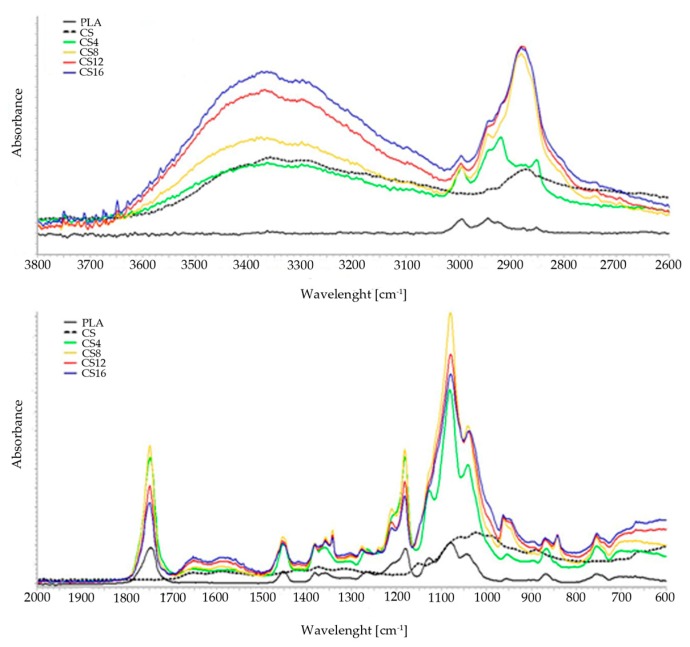
The FT-IR spectra of the PLA/CS foams and the control samples.

The structural properties of the obtained materials were also assessed using the Fourier-transform infrared spectroscopy (FT-IR). The spectra of the PLA, chitosan, and obtained composite foams are shown in Figure 3. The PLA showed the characteristic stretching frequencies for -CH_3_ asymmetric and -CH_3_ symmetric at 2995 cm^−1^ and 2943 cm^−1^, respectively. The -C-H bending frequencies for the same chemical group have been identified at 1451 cm^−1^ and 1383 cm^−1^, respectively [34]. The peak at 1745 cm^−1^ corresponds to the stretching vibration of ester carbonyl group. The next band at 1181 cm^−1^ was ascribed to -C-O- stretching bond in -C-OH- group of the PLA. The specific region composed of three characteristic peaks, ascribed to -C-O- stretching vibration in -O-C=O group, was identified at 1126 cm^−1^, 1080 cm^−1^, and 1041 cm^−1^, respectively [35].

The chitosan spectrum CS demonstrated a broad band in the range of 3600–2900 cm^−1^ attributed to $\upsilon_{\mathrm{NH}}$ and $\upsilon_{\mathrm{OH}}$ vibration and stretching vibrations of -CH_2_- group at 2875 cm^−1^. The peak at 1655 cm^−1^ corresponds to the amide I band. The amide III band located at 1319 cm^−1^ corresponds mainly to the stretching C-N and bending N-H vibration. The saccharide region of the spectrum in the range of 1151–1020 cm^−1^ includes the asymmetric stretching vibration of the C-O-C bridge and the skeletal vibrations involving the C-O-C stretching band [36]. According to the literature data, vibrations characteristic of the PEG molecule are observed at 3400 cm^−1^ (assigned as O-H stretching vibrations of hydroxyl group), C-H stretching of alkanes at 2900 cm^−1^, C-H scissor and bending at 1450–1292 cm^−1^, C-O stretching of alcohol at 1250 cm^−1^, and C-O-C at 1100–1060 cm^−1^ of ether [37,38]. Considering that PLA and chitosan, due to poor miscibility, usually form physical mixtures, no band shifts and no new bands were present in the spectra. It can be stated that there is no new bond formed or strong chemical interaction occurring within the composite foams. The only difference concerns the change in the intensity of the characteristic bands of functional groups present in all polymers used to obtain foams.

### 2.2. Swelling Capacity and Solubility

**Figure 4:**
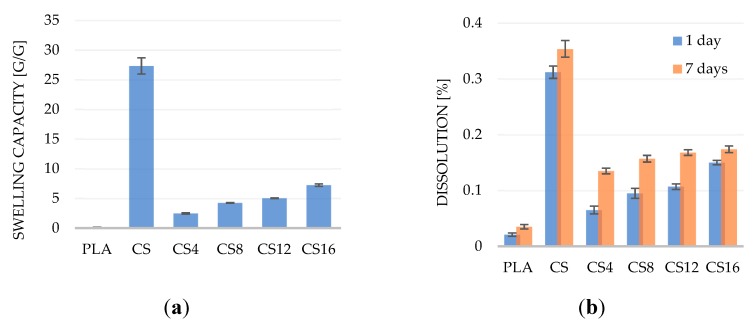
The comparison of (**a**) swelling capacity and (**b**) dissolution of the PLA/CS foams. Values represent means ± standard deviation (*n* = 3, *p* < 0.05).

The results determining the swelling capacity and solubility of the materials in the solution imitating the physiological fluid are presented in Figure 4. The highest swelling capacity was recorded for the control material made only of chitosan CS and it was 27.34 g/g. According to studies by Tiğh and Karakecili [39], the absorbency of sponges made of chitosan is in the range of 27–40 g/g and depends on the degree of polymer deacetylation. This means that 1 g of the polymer can bind from 27 to 40 g of the solution. Dimida and co-workers confirmed that the swelling capacity of chitosan materials is determined by two main factors resulting from the polyelectrolyte nature of the polymer. A larger number of solid polymer charges increase the swelling capacity of the chitosan. However, the increase in the ionic strength of the solution in which the material made of chitosan is incubated results in a decrease in this parameter. This is due to the neutralization of cationic chitosan groups by free ions present in the solution, which reduces the effect of repulsion of polymer chains and swelling of the material caused by osmotic equalization of ion concentration [40]. The control material made of the PLA had the least swelling capacity. In composite foams, swelling capacity increased as the chitosan concentration increased. For the CS16, the sample swelling capacity was 3.8 times lower than for the PLA.

The PLA is not absorbent or soluble in aqueous solutions. However, under such conditions, it is biodegradable, e.g., as a result of the activity of enzymes or microorganisms producing them [41]. The solubility of the obtained materials was low and indirectly resulted from the swelling capacity. The largest weight loss was recorded for the CS sample despite 0.35% dissolution of the total weight of the sample within 7 days. As the concentration of the chitosan in foams increased, their solubility increased but did not exceed the solubility value obtained for the foam made exclusively from the chitosan.

### 2.3. Thermal Properties

**Figure 5:**
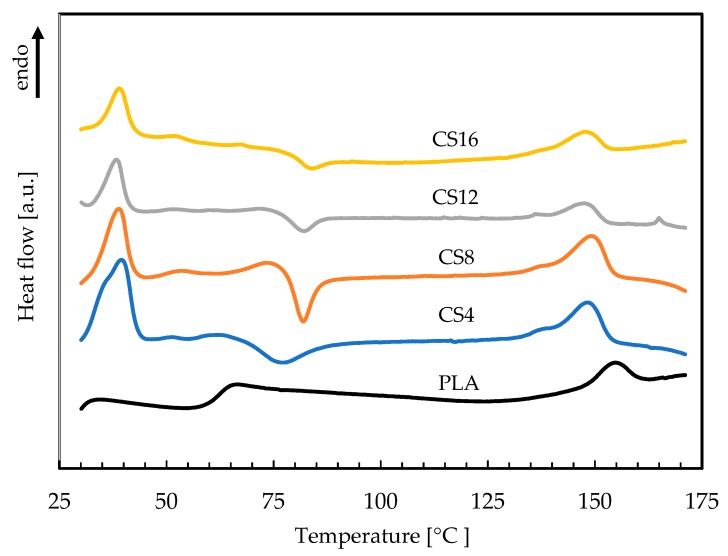
The Differential Scanning Calorimetry (DSC) thermograms of the PLA/CS foams.

The Differential Scanning Calorimetry (DSC) measures the amount of heat energy absorbed or released when the material is heated or cooled. For the polymeric materials, which undergo important property changes near thermal transition, the DSC is a very useful technique to study the glass transition temperature, crystallization temperature, and melting behavior. The DSC curves of the neat PLA and its composites with chitosan are presented in Figure 5 and the determined thermal characteristics are given in Table 2.

All characteristic temperatures of the PLA samples (T_g_, T_c_, T_m_) were similar to those obtained for thermal treated PLA in Carrasco and co-workers’ work [42]. The neat PLA showed two small endothermic peaks. The first was the glass transition temperature (T_g_) and the second was melting temperature (T_m_), respectively. The components of composite foams were three polymers: PLA, PEG, and chitosan. Several authors revealed that the PEG acts as an effective polymeric plasticizer to facilitate the crystallization rates of the PLA [43,44]. Based on the research of Sungsanit and co-workers who used PEG (*M_w_* = 1000 g/mol) to plasticize linear PLA (L-PLA), it can be concluded that glass transition temperature decreased when the PEG content increased, especially since the ability to plasticize PEG is increasing as its molecular weight decreases. [45]. Our research confirmed this theory because the presence of low molecular weight PEG caused a decrease in T_g_ by more than 20 °C. The same researchers showed the crystallization temperature (T_c_) seemed to be increased with increasing PEG content. In our research, the addition of the PEG caused the initiation of crystallization in the obtained foam composites for which the T_c_ value was similar (78–84°C) was due to the equal content of the PEG in materials. A minor decrease in the melting temperature of composite foams with respect to the PLA sample was also observed, indicating that the melting temperature of the PLA was not greatly affected by the addition of the PEG. The addition of chitosan into the PLA polymer led to the minor decrease of the T_g_ and T_m_ (our results during the publication procedure). This is because the chitosan increases the free volume and flexibility of polymeric chains. The addition of chitosan in the PLA matrix can improve its ability to crystallize, which indicates that it can act as a nucleating agent promoting crystallization [46].

### 2.4. Antimicrobial Properties

**Figure 6:**
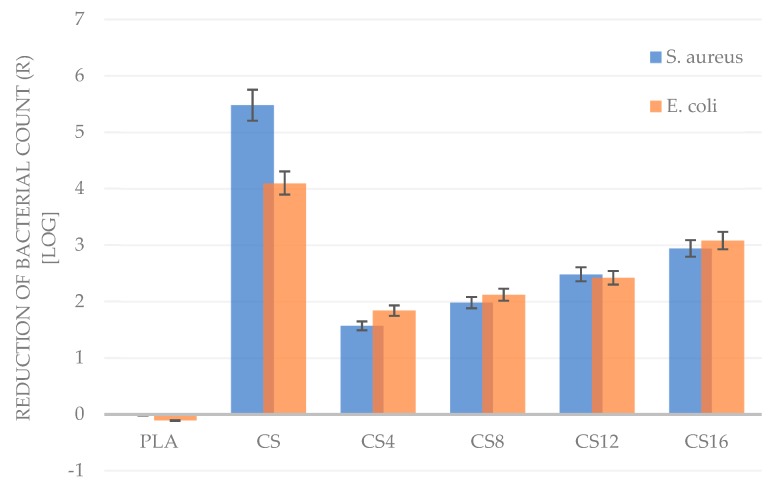
Comparison of the logarithmic reduction of bacteria cells number after incubation with the PLA/CS foams with respect to the K(-) sample after 24 h incubation.

To evaluate the antimicrobial properties of the obtained foams ASTM, E2149 method was used, which is designed to measure the antimicrobial activity of non-leaching antimicrobial surfaces made of plastic, rubber, silicone, and treated fabric material, and is one method to test an irregularly-shaped antimicrobial object [47]. The results presented in Table 3 and Figure 6 indicate that the PLA did not reduce the number of *Escherichia coli* and *Staphylococcus aureus*.

The lack of antimicrobial properties of neat PLA was confirmed also by other authors [48,49]. In the PLA /chitosan composites, activity decreased with decreasing content of chitosan in the foam. Although the foams with a 4% addition of chitosan showed the lowest antimicrobial activity, they caused a reduction in the number of bacteria on a logarithmic scale equal 1.57 and 1.84 for *S. aureus* and *E. coli*, respectively. The highest antimicrobial activity in composite foams noticed for the CS16 sample, for which the logarithmic reduction was 2.94 and 3.08 for *S. aureus* and *E. coli*, respectively. According to the American Society for Microbiology, most of the tested composites present an average reduction of *E. coli* and *S. aureus* strains. The efficient reduction for both strains, determined as a bactericidal effect, was obtained for CS and CS16 samples. Several models suggested that the antimicrobial activity of chitosan results from its cationic nature. The electrostatic interaction between the positively charged polymer and negatively charged microbial cell membranes is predicted to be responsible for cellular lysis and assumed as the main antimicrobial mechanism [18].

### 2.5. Biocompatibility

**Figure 7:**
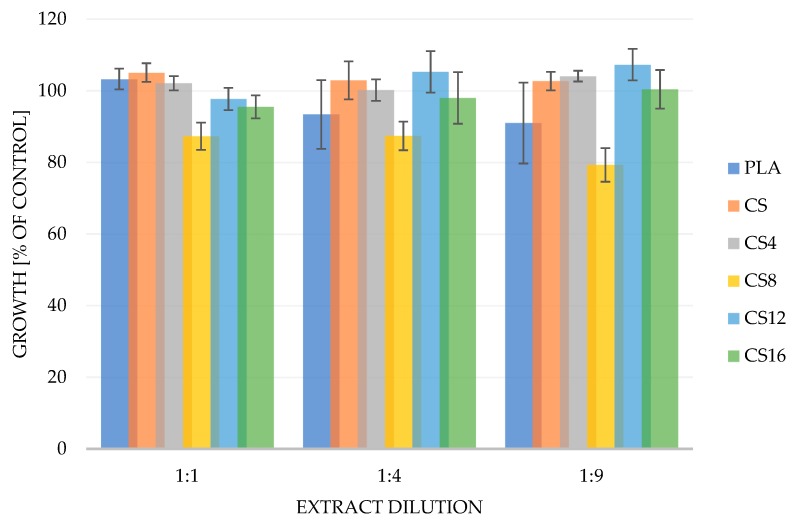
The MTT viability assay of the PLA/CS foams following 24 h of incubation with L929 cells vs. control (untreated) cells. The mean ± standard deviation (SD) values from three independent experiments (*n* = 3; *p* < 0.05).

The in vitro cytotoxicity of the chitosan composite was investigated by the MTT assay using adult mouse fibroblast L929 cells according to the ISO 10993-5:2009(E). This is a standard in vitro method for the biological evaluation of medical devices, which relies on the mitochondrial activity of vital cells and represents a parameter for their metabolic activity [50]. The results are shown in Figure 7 and expressed as the percentage of viable cells versus positive control without the material extracts. The volume dilutions of extracts in medium administered to cells: 1:1, 1:4, and 1:9 correspond to the concentrations: 50 mg/mL, 20 mg/mL, and 10 mg/mL, respectively. It was shown that the maximum inhibition of mouse fibroblast cell growth occurred as a result of treatment with the highest dilution extract for the CS8 sample and did not exceed 20% compared to the control (cells without extract). Considering the occurrence of a possible cytotoxic effect of the tested materials, it should be expected that it would firstly occur in case of the smallest dilution of the extract, where the amount eluted from material components was theoretically the largest. Summarizing the results obtained for all probes and dilutions of their extracts, it can be stated that there was no cytotoxic effect under the condition of this study, which does not preclude their potential use in the production of safe functional biomaterials.

## 3. Materials and Methods

### 3.1. Materials

Medium molecular weight (MMW) chitosan polymer with 75%–85% deacetylation degree (viscosity 200 ÷ 800 cps, 1% concentration solution in 1% acetic acid at 25 °C), phosphate buffer saline (PBS), and poly(ethylene glycol) (PEG; molecular weight = 400 g/mol) were purchased from Sigma-Aldrich (Saint Louis, MO, USA). The PLA pellets were purchased from ORBI-TECH (Leichlingen, Germany). Calcium chloride, lactic acid, sodium hydroxide, sodium chloride, and chloroform were purchased from “Avantor Performance Materials Poland” (Gliwice, Poland). Carbon dioxide was bought from “Linde” (Gdansk, Poland).

The bacterial strains: *Escherichia coli* K-12 PCM 2560 (NCTC 10538) and *Staphylococcus aureus* PCM 2054 (ATCC 25923) were provided from Polish Collection of Microorganisms, Ludwik Hirszfeld Institute of Immunology and Experimental Therapy of the Polish Academy of Sciences, Wrocław, Poland. The TSB, TSA, and peptone were purchased from “Biocorp” (Warsaw, Poland). The 3-(4,5-dimethylthiazol-2-yl)-2,5-diphenyltetrazolium bromide (MTT), medium, antibiotics, and supplements necessary for cell culture were obtained from Sigma-Aldrich (St. Louis, MO, USA). MilliQ water was used for the preparation of all aqueous solutions. All other reagents were of analytical grade or higher.

### 3.2. Foams Preparation

In the first stage, the 9% (*w*/*v*) PLA solution was prepared. The 90 g of PLA granulate was suspended in 910 mL of chloroform in a sealed bottle and dissolved at 30 °C for 24 h with continuous stirring (Thermo Scientific Forma Orbital Shaker, Marietta, OH, USA). The 1% *w*/*w* chitosan solution (CS) was prepared by using a method of dissolving an aqueous chitosan suspension with gaseous CO_2_ [32]. The 15 g of chitosan was dissolved in 985 g of 0.1 M lactic acid and then 0.5 M sodium hydroxide was slowly added with constant agitation until a pH of 8.5 was reached. In the next step, chitosan precipitate was separated by centrifugation: 9000× *g*, 20 min (MPW-350R, Warszawa, Poland) washed three times with distilled water, and centrifuged again. The chitosan precipitate was weighed and added to distilled water, so that the final mass of the mixture, together with the precipitate, had a mass of 1500 g. The solution was saturated with CO_2_ until fully dissolved with the use of an analog overhead stirrer (BIOMIX BMX-10, Gdańsk, Poland). The PLA and the CS solutions were combined with the addition of the polyethylene glycol (PEG) and stirred mechanically to obtain a homogeneous mixture. The mixture inserted in silicone form, frozen, and freeze-dried (Christ Alpha 1–4 LD Plus; 0.94 mbar, sample temperature: 20 °C, condenser temperature: −50 °C). The composition of the foams used in experiments is shown in Table 4.

### 3.3. Foams Characterization

#### 3.3.1. Topography Evaluation

Topography evaluation was carried out using variable-pressure scanning electron microscopy VP-SEM S3400-N (Hitachi, Hyogo, Japan) equipped with a tungsten source. The accelerating voltage was 20 kV. The charge compensation was assured through conducting micrographs at 120 Pa and BSE detector. The pictures of filament fragments were taken with a Nikon D7200 camera (Kumagaya, Japan).

#### 3.3.2. Porosity and Density Measurements

The porosity and density were determined via a liquid displacement method with ethanol as the displacement liquid because it easily penetrates the pores of the foams and does not induce shrinkage or swelling as a nonsolvent of the polymers according to methods described by Zhang and Ma [51]. A foam sample of weight W was immersed in a graduated cylinder containing a known volume (V_1_) of ethanol. The sample was kept in the ethanol for 5 min and then a series of brief evacuation–repressurization cycles were conducted to force the ethanol into the pores of the foam. Cycling was continued until no air bubbles were observed emerging from the foam. The total volume of ethanol and the ethanol-impregnated foam then was recorded as V_2_. The volume difference (V_2_ − V_1_) was the volume of the skeleton of the foam. The ethanol-impregnated foam was removed from the cylinder and the residual ethanol volume recorded as V_3_. The quantity (V_1_ − V_3_)—the volume of the ethanol held in the foam—was determined as the void volume of the foam. Thus, the total volume of the foam was: V= (V_2_ − V_1_) + (V_1_ − V_3_) = V_2_ − V_3_. The density of the foam (d) was expressed as:

*d* = W/(V_2_ − V_3_) (1)

and the porosity of the foam (P) was obtained by:

P = (V_1_ − V_3_)/(V_2_ − V_3_) (2)

#### 3.3.3. Fourier-transform Infrared Spectroscopy (FT-IR) Study

The FT-IR spectra were measured using the FT-IR spectrometer (Nicolet 8700; Thermo Electron Corp., Waltham, MA, USA) equipped with the GoldenGate (Specac Corp., Orpington, UK) ATR accessory with a single reflection diamond crystal. The temperature of the crystal was maintained at 25.0 ± 0.1 °C by using an automatic temperature controller (Specac Corp, Orpington, UK) coupled with the ATR accessory. In each measurement, 64 scans were collected with a resolution of 4 cm^−1^ and the range of 4000–550 cm^−1^. The spectrum of the filament was measured and later subtracted from every measured spectrum as the background. After measuring all FT-IR spectra corresponding to a selected strain and background subtraction, the average spectrum was calculated. The spectrometer was purged with dry nitrogen to diminish the negative influence of water vapor. Spectragryph V1.2.10 software (Oberstdorf, Germany) was used to process the obtained spectra.

#### 3.3.4. Swelling Capacity/Dissolution Evaluation

The swelling capacity of the foams was evaluated by gravimetric determination of the foams before (W) and after (W_1_) placing the foams in an aqueous solution containing a salt composition similar to that of wound exudate according to EN 13726-1, prepared by adding 8.298 g of NaCl and 0.368 g of CaCl_2_ × 2H_2_O to 1 L of distilled water. The samples were incubated at 37 °C for 24 h, withdrawn from the medium, and weighed after removal of the surface fluid using a filter paper. The samples were then freeze-dried (CHRIST Alpha 1–4 LD plus, Osterode am Harz, Germany) and weighed again (W_2_). The fluid absorption was defined as the ratio of the weight increase (W_1_–W_2_) relative to the weight remaining after freeze-drying (W_2_). Each value was calculated as the mean of three independent measurements. The dissolution of the foam (%) was defined as the ratio of the weight decrease of the foam (W − W_2_) relative to the initial weight (W).

#### 3.3.5. Mechanical Properties

The mechanical properties of the foams were characterized using a universal testing machine (Instron model 5543, controlled using the “Merlin” software V 4.42. Warszawa, Poland) according to previously described methods [52,53]. Five cylindrical samples of the test foams (Ø 25 mm × 4 mm) were compressed up to 50% deformation at a test speed of 0.5 mm/s and the compression load (kPa) was measured. Based on the obtained results, the texture profile values were determined: hardness and flexibility.

#### 3.3.6. Thermal Properties

The study of thermal analysis was carried out with the DSC Diamond Perkin Elmer (Norwalk, CT, USA), which was calibrated against an indium standard in a nitrogen atmosphere (flow rate of 20 mL/min). An empty aluminum pan was the reference. The weight of the samples used for the determinations was 10 ± 2 mg. The DCS procedure consisted of three steps. At the first step, the samples were heated from 25 °C to 200 °C with a heating rate of 10 °C/min, then they were cooled to 25 °C, at cooling rate of 10 °C/min. In the last step, they were reheated to 200 °C at a heating rate of 10 °C/min. From the DSC thermograms (second run), we studied the effect of chitosan on the thermal properties of the PLA such as glass transition temperature (Tg), exothermic crystallization temperature (Tc), and endothermic melting temperature (Tm) [34].

#### 3.3.7. Antimicrobial Properties

The evaluation of antimicrobial properties of filaments was made according to the quantitative ASTM E2149 method with slight modification using *E. coli* and *S. aureus* strains [47]. The strains were deep-frozen and stored in CrioBanks; their biochemical features were regularly controlled and, before the test, they were multiplied on nutrient agar. The 1 g of foam previously cut into 2 mm fragments was put into the 250 mL conical flask and flooded with 0.3 mM KH_2_PO_4_ solution with bacteria at a density of 3.0 × 10^5^ cfu/mL. Samples in two repetitions and a reference without a sample were incubated for 24 h at 37 °C (Heidolph Incubator 1000, Merck Sp. z o.o., Warszawa, Poland) with constant shaking at 200 rpm (Heidolph Unimax 1010, Merck Sp. z o.o., Warszawa, Poland). The 1 mL of the suspension was collected from each of the flasks and the number of bacteria (cfu/mL) was estimated by the method of decimal dilution on nutrient agar. After incubation, plates with 15–300 cfu were counted. When there were no colonies on the plate, the number was recorded as “10”. Incubation was made from either dilution onto two parallel plates. The number of viable counts (cfu/mL) was established according to the following formula:

*V_c_* = N × D (3)

where *V_c_* is the bacteria concentration in colony forming units in milliliter (cfu/mL), N is the average value of bacteria counted from Petri dishes in colony forming units in milliliter (cfu/mL), and D is the dilution factor from the counted Petri dish.

The result of the analysis presented as a reduction in the number of bacteria calculated from the formula:

*R* = log (B/A) (4)

where A is the average of the number of viable cells on the test sample after 24 h incubation at 37 °C and B is the average of the number of viable cells on the control sample after 24 h incubation at 37 °C.

The percentage reduction of bacteria/fungi on a logarithmic scale (R) equal 1, 2, and 3 corresponds to a reduction of 90%, 99%, and 99.9%, respectively.

#### 3.3.8. Biocompatibility Tests

##### Cell Culture

The L929 cells (adult mouse fibroblast cell line) were obtained from ATCC (American Type Culture Collection, Manassas, VA, USA). The cells (mycoplasma free) were maintained in monolayer culture at 37 °C in a humidified 5% CO_2_ atmosphere in Low Glucose Dulbecco’s modified Eagles’ medium (DMEM) supplemented with 10% fetal bovine serum (FBS), 100 units/mL penicillin, 100 μg/mL streptomycin, and 10 mM glutamine (Sigma-Aldrich, St. Louise, CA, USA). Under these conditions, the cell-doubling time was 24 h.

##### Sample Preparation

The sample preparation was carried out according to the ISO 10993-12:2002 standard. The foam samples weighing 0.5 ± 0.01 g were cut into small pieces and placed within clean, chemically inert, and sterile containers with minimum headspace with 5 mL of the phosphate-buffered saline (PBS) solution and closed. The extractions were performed at 37 ± 1 °C for 72 ± 2 h with agitation.

##### Cell Viability

The cytotoxicity of the foams was assessed by 3-(4,5-dimethylthiazol-2-yl)-2,5-diphenyltetrazolium bromide (MTT) assay according to the ISO 10993-5:2009(E). The L929 cells at a density of 1 × 10^4^ cells/well were seeded on 96-well tissue culture microtiter plate and 100 μL culture medium only (blank) was dispensed into peripheral wells. The cells were incubated for 24 h (5% CO_2_, 37 °C, >90% humidity) to form a half-confluent monolayer. After 24 h incubation, the culture medium was aspirated from the cells and 100 μL of treatment medium containing either the appropriate concentration of sample extracts or the positive control or blank was added. After 24 h of incubation, the cell viability and morphology were examined. Then, attached and proliferated cells were quantified using the MTT assay. Briefly, the culture medium was removed and 50 μL of the MTT solution (1 mg/mL in medium without supplements and phenol red) was added to each well and incubated for 2 h at 37 °C in a humidified 5% CO_2_ atmosphere. After that, the media with the MTT solution was removed and crystals of formazan from each well were suspended in 100 μL of isopropanol. The plate was left on the shaking platform for 10 min. The absorbance was recorded on a Microplate Reader (Bio-Rad, Hercules, CA, USA) at the length of 570 nm. The experiments were repeated at least three times. A decrease in the number of living cells correlates to the amount of blue-violet formazan formed, as monitored by the optical density at 570 nm. To calculate the reduction of viability compared to the blank, the equation was used:

*Viability* (%) = 100 × OD_570e_/OD_570pc_ (5)

where OD_570e_ is the mean value of the optical density of the samples and OD_570pc_ is the mean value of the optical density of the positive control (the L929 cells in culture medium).

#### 3.3.9. Statistical Analyses

The STATISTICA software (StatSoft, Inc., Tulsa, OK, USA) was used for analyses. The statistical significance was determined at *p* < 0.05. All data reported were based on the means of three replicates (*n* = 3). Experimental results were expressed as mean ± standard deviation (SD). Student’s t-test and one-way analysis of variance (ANOVA) were applied. The differences were considered to be statistically significant at *p* < 0.05.

## 4. Conclusions

The performed study proved that the use of CO_2_ saturation of the chitosan precipitate suspension method is suitable for obtaining functional foamed materials with PLA. The method is simple and does not require additional steps to remove acids from the finished material, which is usually used to dissolve chitosan polymer. The obtained foams were characterized by a lack of cytotoxicity toward L929 fibroblast cells, low density, and high porosity and hardness. The addition of chitosan provides also the functional properties of sponges in the form of antimicrobial activity. All of PLA/CS materials limited bacterial growth of *S. aureus* and *E. coli*, but the best bactericidal effect was obtained when the concentration of chitosan in the foam was 16%. Research confirms that these types of functional materials could be successfully used when not only is the bactericidal needed, but also bacteriostatic effect in the bioproduction sector.

## Figures and Tables

**Table 1:** The density and porosity of the polylactic acid/chitosan (PLA/CS) foams. Values represent means ± standard deviation (*n* = 3, *p* < 0.05).

Sample	Density [kg/m^3^]	Porosity [%]
PLA	1250 ± 6 ^a^	Nd.
CS	49 ± 7 ^b^	80.4 ± 2.2 ^A^
CS4	168 ± 8 ^c^	53.1 ± 2.0 ^B^
CS8	92 ± 10 ^d^	60.5 ± 3.1 ^C^
CS12	69 ± 9 ^d^	73.9 ± 3.4 ^D^
CS16	55 ± 11 ^b^	85.3 ± 2.7 ^A^

Nd.—Not detected. Values marked with the same letters in column do not differ significantly. Lowercase letters—against the PLA control sample. Capital letters—against the CS control sample.

**Table 2:** The characteristic thermal transition temperatures of the obtained filaments.

Sample	Tg [°C]	Tc [°C]	Tm [°C]
PLA	65.7	-	156.3
CS4	41.9	77.6	149.8
CS8	39.7	82.1	150.5
CS12	39.0	82.4	148.6
CS16	39.6	84.1	149.2

**Table 3:** Comparison of the average number of bacterial cells after incubation with the PLA/CS foams.

Sample	Time of Interaction [h]	Average Number of Cells [cfu/mL]	
		*S. Aureus*	*E. Coli*
**K(-)**	0	1.16 × 10^5^	2.36 × 10^5^
K(-)	24	3.12 × 10^7^	1.54 × 10^7^
PLA	24	3.31 × 10^7^	2.00 × 10^7^
CS	24	1.02 × 10^2^	1.23 × 10^3^
CS4	24	8.45 × 10^5^	2.20 × 10^5^
CS8	24	3.25 × 10^5^	1.16 × 10^5^
CS12	24	1.03 × 10^5^	5.85 × 10^4^
CS16	24	3.56 × 10^4^	1.28 × 10^4^

K(-)—Negative control sample with no antimicrobial activity determined by ASTM E2149.

**Table 4:** Composition of foams.

Sample	Concentration of Polymer in Foam [% *w*/*w*]		
	PLA	Chitosan	PEG
PLA	100	0	0
CS	0	100	0
CS4	73	4	23
CS8	69	8	23
CS12	65	12	23
CS16	61	16	23
